# Curcumin versus placebo in benign prostatic hyperplasia: a systematic review and meta-analysis

**DOI:** 10.1007/s00228-026-04093-9

**Published:** 2026-06-05

**Authors:** Diogo Souto Santana, Breno Cordeiro Porto, Rafael Baldissera Cardoso, Guilherme Melchior Maia Lopes, Luiz Guilherme Serrão Gimenez, Carlo Camargo Passerotti, Ronaldo Maia, Fernando Gonçalves Almeida, Rodrigo Afonso da Silva Sardenberg, José Arnaldo Shiomi da Cruz

**Affiliations:** 1 University Centre FMABC – Santo Andre, São Paulo, Brazil; 2 Research Institute - Hapvida NotreDame, São Paulo, Brazil; 3 Moinhos de Vento Hospital - Porto Alegre, Rio Grande Do Sul, Brazil; 4 Universidade de São Paulo - School of Medicine, São Paulo, Brazil; 5 Department of Urology, Escola Paulista de Medicina, Federal University of São Paulo, São Paulo, Brazil; 6 Ninth of July University, São Bernardo do Campo, Brazil

**Keywords:** Curcuma, Prostatic hyperplasia, Systematic review, Meta-analysis

## Abstract

**Background:**

Benign prostatic hyperplasia (BPH) is a highly prevalent condition among aging men and a major cause of lower urinary tract symptoms (LUTS). Although α1-adrenoceptor blockers and 5-alpha-reductase inhibitors remain the cornerstone of treatment, their long-term use is frequently limited by adverse effects and suboptimal symptom control. Growing experimental and clinical evidence suggests that chronic prostatic inflammation plays a central role in BPH progression, raising interest in anti-inflammatory compounds such as curcumin. However, the clinical effectiveness of curcumin in BPH remains poorly defined.

**Methods:**

We conducted a systematic review and meta-analysis of randomized and nonrandomized comparative studies evaluating curcumin versus placebo in men with BPH receiving α-blockers and/or 5-alpha-reductase inhibitors. Searches were performed in PubMed, Embase, and Cochrane CENTRAL. The primary outcome was change in International Prostate Symptom Score (IPSS). Secondary outcomes included prostate-specific antigen (PSA), prostate volume (PV), post-void residual volume (PVR), and maximum urinary flow rate (Q-max). Pooled analyses were performed using a random-effects model.

**Results:**

Six studies involving 697 patients met the inclusion criteria. Compared with placebo, curcumin was associated with a significant improvement in IPSS (MD − 4.11; *p* = 0.0009), accompanied by reductions in PSA (MD − 0.52 ng/mL), PV (MD − 3.78 mL), and PVR (MD -2.38 mL), as well as an increase in Q-max (MD 2.09 mL/s). Subgroup analyses suggested greater symptomatic benefit among patients receiving α-blockers alone compared with those on combined α-blocker and 5-alpha-reductase inhibitor therapy. Substantial heterogeneity was observed across outcomes.

**Conclusion:**

Curcumin supplementation was associated with significant improvement in LUTS and functional parameters in men with BPH. Although these findings support a potential adjunctive role for curcumin, the available evidence is limited by heterogeneity and study design variability. Well-designed, large-scale randomized trials are warranted to define its clinical utility, optimal formulation, and long-term effects.

**Supplementary Information:**

The online version contains supplementary material available at 10.1007/s00228-026-04093-9.

## Introduction

Benign prostatic hyperplasia (BPH) represents one of the most common chronic conditions affecting aging men and constitutes a leading cause of lower urinary tract symptoms (LUTS) worldwide [1, 2]. Epidemiological data indicate that histological evidence of BPH is present in more than 50% of men over the age of 60 and in up to 80% of those older than 80 years. Clinically, progressive prostate enlargement and bladder outlet obstruction are frequently accompanied by urinary frequency, nocturia, urgency, weak stream, and incomplete emptying, symptoms that significantly impair quality of life and impose a substantial socioeconomic burden [3].

Pharmacological management of BPH is primarily based on α1-adrenoceptor blockers (AB) and 5-alpha-reductase inhibitors (5-ARI) [4]. While α-blockers rapidly improve urinary flow by reducing smooth muscle tone, 5-ARIs act by inhibiting dihydrotestosterone synthesis, leading to gradual prostate shrinkage and reduced risk of progression. Despite their established efficacy, these therapies are often limited by adverse effects, including ejaculatory dysfunction, decreased libido, erectile dysfunction, dizziness, and hypotension, which frequently compromise adherence. Discontinuation rates due to adverse events have been reported to range from approximately 5% to 15% for α-blockers and up to 15–25% for 5-alpha-reductase inhibitors in long-term studies, particularly due to sexual dysfunction and reduced quality of life [4, 5].

Over the past decade, increasing attention has been directed toward the role of chronic inflammation in the pathophysiology of BPH [6]. Histological studies consistently demonstrate inflammatory infiltrates in hyperplastic prostate tissue, and inflammatory mediators such as interleukin-6, interleukin-8, tumor necrosis factor-alpha, and cyclooxygenase-2 have been implicated in prostatic stromal proliferation, tissue remodeling, and symptom severity [7]. These findings support the concept that BPH is not merely a hormonally driven proliferative disorder but also an inflammatory disease, providing a strong biological rationale for anti-inflammatory therapeutic approaches.

Curcumin, a polyphenolic compound derived from Curcuma longa, exhibits well-documented anti-inflammatory, antioxidant, and antiproliferative properties. Experimental models have demonstrated that curcumin downregulates NF-κB signaling, suppresses pro-inflammatory cytokine expression, and modulates growth factor pathways involved in prostatic hyperplasia [8, 9]. In animal models of testosterone- or inflammation-induced BPH, curcumin administration has been associated with reduced prostate volume and attenuation of inflammatory markers [8, 9]. These biological effects have driven growing clinical interest in curcumin-based formulations as potential adjuncts in the management of BPH-related LUTS.

Nevertheless, despite the increasing availability of curcumin supplements and emerging clinical trials, its role in BPH management remains poorly defined. Major urological guidelines, including those of the European Association of Urology (EAU) and the American Urological Association (AUA), do not currently provide specific recommendations regarding curcumin use, reflecting the absence of consolidated high-level evidence [10, 11]. Existing clinical studies are heterogeneous, encompassing different curcumin formulations, dosages, follow-up durations, and background pharmacological therapies, and their results have not been systematically synthesized.

Therefore, the present study aimed to perform a comprehensive systematic review and meta-analysis of comparative studies evaluating curcumin versus placebo in men with BPH receiving standard medical therapy. By integrating clinical, biochemical, and functional outcomes, this analysis seeks to clarify the potential efficacy of curcumin supplementation, explore differential effects according to background therapy, and critically appraise the current evidence base to inform future research and clinical practice.

## Materials and methods

### Study design and reporting standards

This systematic review and meta-analysis was conducted in accordance with the Cochrane Collaboration Handbook for Systematic Reviews of Interventions and is reported following the Preferred Reporting Items for Systematic Reviews and Meta-Analyses (PRISMA) statement [12]. The study protocol was prospectively registered in the International Prospective Register of Systematic Reviews (PROSPERO) under the number CRD42024555920.

### Search strategy

A comprehensive literature search was performed in PubMed (MEDLINE), Embase, and the Cochrane Central Register of Controlled Trials (CENTRAL) from inception to May 27, 2025. The search strategy combined Medical Subject Headings (MeSH) and free-text terms related to curcumin and benign prostatic hyperplasia, including: (“Curcumin”[MeSH] OR curcumin* OR “Curcuma longa” OR curcuma) AND (“Prostatic Hyperplasia”[MeSH] OR “benign prostatic hyperplasia” OR BPH OR “benign prostatic enlargement” OR “lower urinary tract symptoms” OR LUTS) AND (“International Prostate Symptom Score” OR IPSS OR prostate). Searches were complemented with methodological filters for clinical trials when appropriate.

No language restrictions were applied. In addition, the reference lists of all included studies and relevant review articles were manually screened to identify potentially eligible publications not captured by the initial search.

### Eligibility criteria

Studies were considered eligible if they met all of the following criteria: (1) comparative clinical studies, including randomized controlled trials (RCTs) and nonrandomized studies; (2) adult male patients diagnosed with benign prostatic hyperplasia; (3) comparison between curcumin (or curcumin-based formulations) and placebo; (4) concomitant treatment with α1-adrenoceptor blockers and/or 5-alpha-reductase inhibitors permitted; (5) minimum follow-up duration of three months; and (6) reporting of the International Prostate Symptom Score (IPSS) total score.

Studies were excluded if they: (1) included patients receiving anticholinergic agents; (2) involved surgical or minimally invasive interventions for BPH; (3) had a follow-up shorter than three months; (4) were case reports, narrative reviews, editorials, or conference abstracts without sufficient data.

### Study selection

**Fig. 1:**
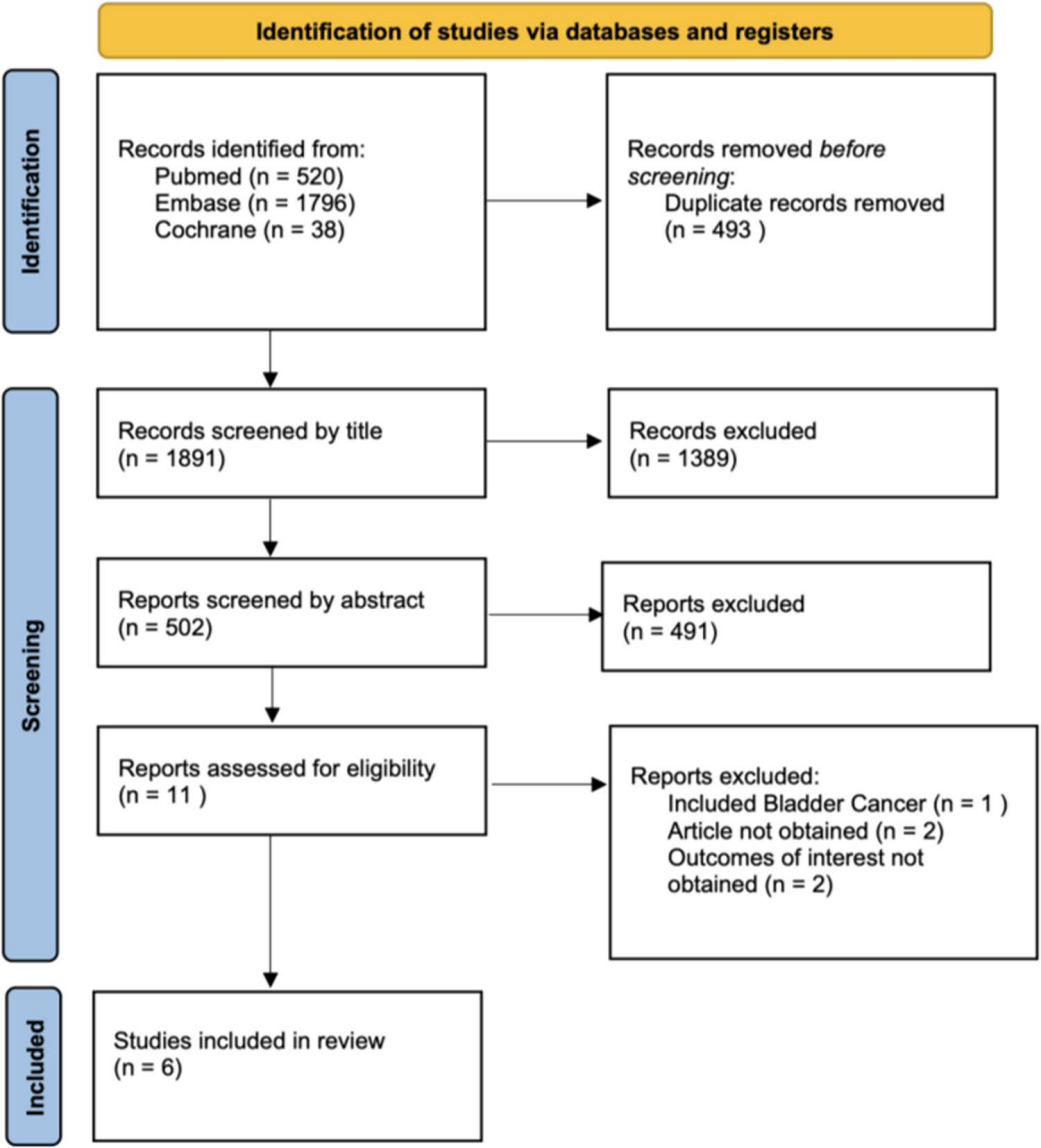
PRISMA flow diagram of study selection process

After removal of duplicates, two reviewers independently screened titles and abstracts for potential eligibility. Full texts of potentially relevant studies were subsequently assessed for inclusion. Disagreements were resolved through discussion and, when necessary, consultation with a third reviewer. The study selection process is summarized in the PRISMA flow diagram (Fig. 1).

### Data extraction

Data extraction was independently performed by two investigators using a standardized, predefined data collection form. Extracted variables included first author, year of publication, country, study design, sample size, patient characteristics, type and dose of curcumin formulation, background pharmacological therapy, follow-up duration, and outcome measures. Information regarding whether curcumin was administered as monotherapy or as part of combination nutraceutical formulations was also extracted. When available, data on IPSS quality-of-life (QoL) scores were also extracted.

For continuous outcomes, means, standard deviations, and sample sizes were collected for both intervention and control groups. When data were incomplete or unclear, attempts were made to derive the necessary values from the published material.

### Endpoints and subgroup analyses

The primary endpoint was change in International Prostate Symptom Score (IPSS) total score.

Secondary endpoints included changes in: prostate-specific antigen (PSA) levels (ng/mL), prostate volume (PV, mL), post-void residual volume (PVR, mL), and maximum urinary flow rate (Q-max, mL/s).

Prespecified subgroup analyses were performed according to background medical therapy: (1) α1-adrenoceptor blockers alone, and (2) combination therapy with α1-adrenoceptor blockers and 5-alpha-reductase inhibitors.

An additional exploratory subgroup analysis was planned for treatment-naïve patients when data were available.

### Risk of bias assessment

Risk of bias in randomized controlled trials was evaluated using the Cochrane Risk of Bias tool for randomized trials (RoB-2) [13]. Nonrandomized studies were assessed with the Risk Of Bias In Non-randomized Studies of Interventions (ROBINS-I) tool [14].

**Fig. 2:**
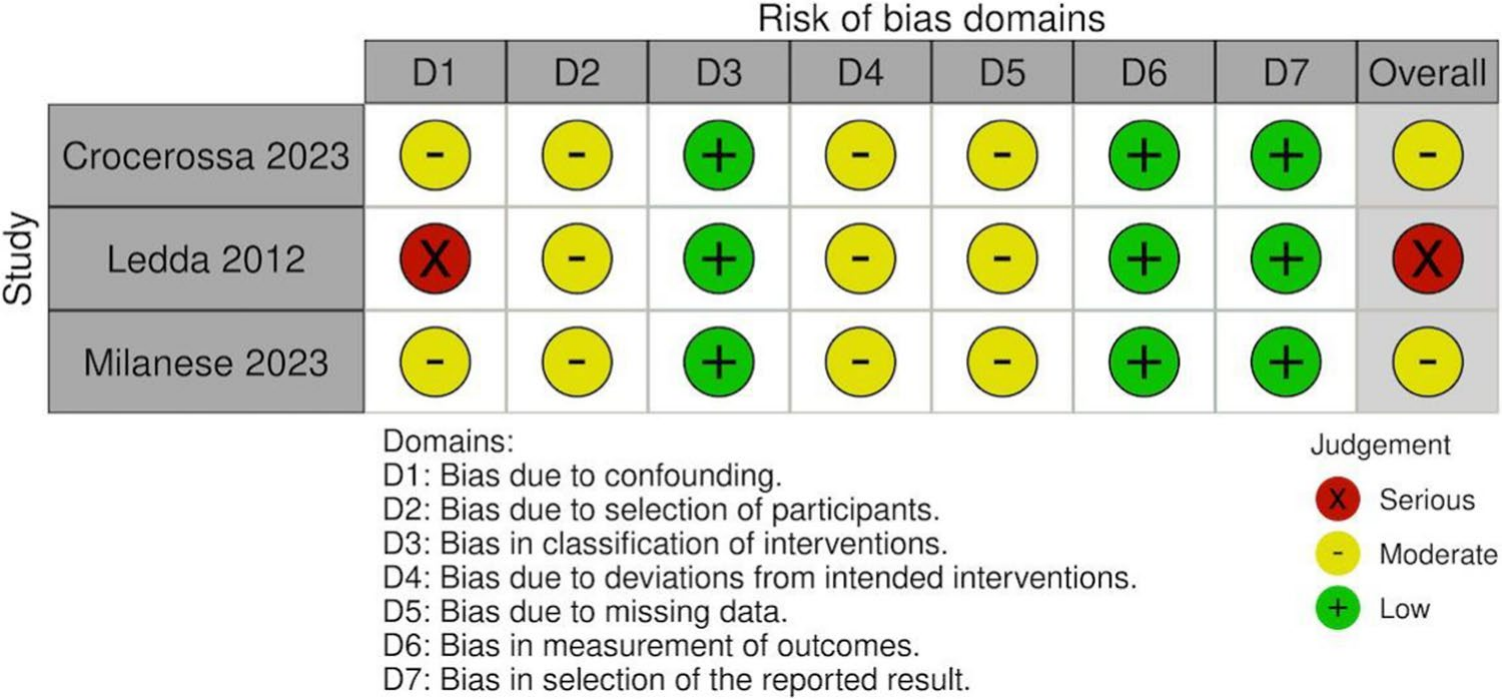
Risk of bias summary for non-randomized studies (ROBINS-I tool)

**Fig. 3:**
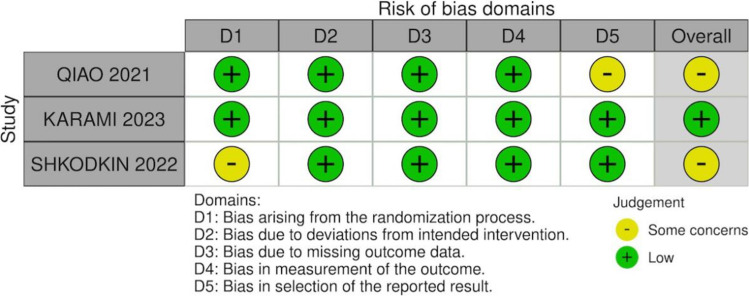
Risk of bias summary for randomized controlled trials (RoB 2 tool)

Two reviewers independently performed all risk-of-bias assessments. Discrepancies were resolved by consensus after joint review. For Risk-of-bias plots, please refer to Figs. 2 and 3.

### Statistical analysis

Continuous outcomes were summarized using mean differences (MDs) with corresponding 95% confidence intervals (CIs). Pooled estimates were calculated using a random-effects model, considering the expected clinical and methodological heterogeneity among studies.

Statistical heterogeneity was assessed using Cochran’s Q test and quantified with the I^2^ statistic, and performed with RStudio: Integrated Development for R. RStudio, PBC, Boston, MA [15]. Heterogeneity was considered substantial when I^2^ exceeded 50%.

Leave-one-out sensitivity analyses were conducted for the primary endpoint to evaluate the robustness of the pooled estimates. Prespecified subgroup analyses were performed based on background pharmacological therapy.

All statistical analyses were conducted using Review Manager (RevMan) version 5.4 (The Cochrane Collaboration, Copenhagen, Denmark) [16]. A two-sided p-value < 0.05 was considered statistically significant.

## Results

### Study selection and characteristics

The systematic search identified a total of 2,354 records. After removal of duplicates and screening of titles and abstracts, 11 articles were retrieved for full-text assessment. Of these, six studies fulfilled the eligibility criteria and were included in the final analysis [17–22]. The reasons for exclusion are detailed in the PRISMA flow diagram (Fig. 1).

The six included studies comprised a total of 697 participants, of whom 353 (50.6%) received curcumin-based supplementation and 344 (49.4%) received placebo. Three studies were randomized controlled trials and three were nonrandomized comparative studies. Follow-up duration ranged from 3 to 12 months. Considerable variability was observed in curcumin formulations, dosages, and background pharmacological therapy. In most included studies, curcumin was administered as part of enhanced-bioavailability formulations or in combination with other compounds, which may influence pharmacokinetics and clinical outcomes. Detailed baseline characteristics of the included studies are summarized in Table 1. Detailed outcome data are presented in Table 2.

**Table 1:** Baseline characteristics of included studies

Study	Design	Population	Intervention	Control	Age (years) Mean ± SD—Curmin/Placebo	N.º of patients—Curmin/Placebo	Follow-up in months
Crocerossa 2023 A (Italy)	Prospective Non-RCT	Naive	Curcumin (75 mg daily)	Placebo	66.2 (8.2)/67.6 (7.3)	76/76	6
Crocerossa 2023 B (Italy)	Prospective Non-RCT	AB	Curcumin (75 mg daily)	Placebo	67.1 (9.4)/67.6 (9.2)	69/69	6
Crocerossa 2023 C (Italy)	Prospective Non-RCT	AB + 5-ARI	Curcumin (75 mg daily)	Placebo	71.5 (3.5)/71.7 (3)	39/39	6
Karami 2023 (Iran)	RCT	AB + 5-ARI	Curcumin (160 mg/daily)	Placebo	61.5 (7.7)/60.1 (6.9)	25/25	3
Ledda 2012 (Italy)	Prospective Non-RCT	AB + 5-ARI	Curcumin (100 mg/daily)	Placebo	58.6 (5.3)/58.3 (3.4)	33/28	6
Milanese 2023 (Italy)	Prospective Non-RCT	AB	Curcumin (1,200 mg/daily)	Placebo	68.0 (4.0)/65.5 (4)	20/20	12
Qiao 2021 (China)	RCT	AB + 5-ARI	Curcumin (2,250 mg/daily)	Placebo	60.8 (5.0)/59.3 (5.3)	61/61	6
Shkodkin 2022 (Russia)	RCT	AB	Curcumin (400 mg/daily)	Placebo	63.5 (2.9)/64.4 (5.2)	30/26	12

*AB* alpha-blocker, *5-ARI* 5alpha reductase inhibitors, *RCT* Randomized Controlled Trial

**Table 2:** Summary of outcomes and quantitative data extracted from included studies

Study	N.º of patients—Curmin/Placebo	PSA(ng/mL) Mean ± SD—Curmin/Placebo	PV (mL) Mean ± SD—Curmin/Placebo	IPSS Mean ± SD—Curmin/Placebo	Q max (ml) Mean ± SD—Curmin/Placebo	PVR (mL) Mean ± SD—Curmin/Placebo
Crocerossa 2023 A (Italy)	76/76	2.7 (1.4)/2.6 (1.2)	61.3 91(6.8)/63 9 (20.3)	19.5 (4.1)/19.4 (3.6)	10.2 (3.1)/10.5 (2.9)	63.3 (22.6)/62 (24.4)
Crocerossa 2023 B (Italy)	69/69	2.5(1.3)/2.5 (1)	60.3 (20.9)/58.9 (24.4)	21.7 (3.6)/21.1 (3.3)	11.2 (2.8)/10.9 (2.4)	55.5 (25.6)/58.9 (27.1)
Crocerossa 2023 C (Italy)	39/39	2.7 (0.8)/2.5 (0.8)	65.4 (26.7)/67.5 (27.3)	21.7 (3)/21.5 (3.5)	8.8 (2.5)/9 (2.8)	71.5 (28.9)/70 (26.1)
Karami 2023 (Iran)	25/25	6.3 (1.3)/6.0 (1.1)	29.1 (2.1)/29.3 (2.2)	10.3 (3.3)/10.4 (2.8)	NI/NI	NI/NI
Ledda 2012 (Italy)	33/28	5.7 (0.2)/5.64 (0.1)	NI/NI	27.98/28.03	NI/NI	NI/NI
Milanese 2023 (Italy)	20/20	NI/NI	56 (18)/52 (13)	15 (5)/15.5 (8)	15.75 (2.9)/16.6 (3.3)	55 (15)/62.5 (18)
Qiao 2021 (China)	61/61	2.12(0.7)/2.27 (1.0)	49.2 (14.8)/45.9 (13.6	17.2 93.8)/18.3 (3.67)	9.03 (2.84)/9.26 (2.79)	NI/NI
Shkodkin 2022 (Russia)	30/26	4.1 (1.6)/6.2 (2.5)	65.3 (12)/72.5 (24.1)	23.8 (2.5)/23.7 (3.0)	9.7 (1.3)/10.5 (2.1)	64.4 (11)/79.1 (27.2)

*SD* Standard Deviation, *PSA* prostate specific antigen, *PV* prostate volume, *IPSS* international prostate symptom score, *PVR* post-voided residual volume, *NI* Not Informed

### Meta-analysis

**Fig. 4:**
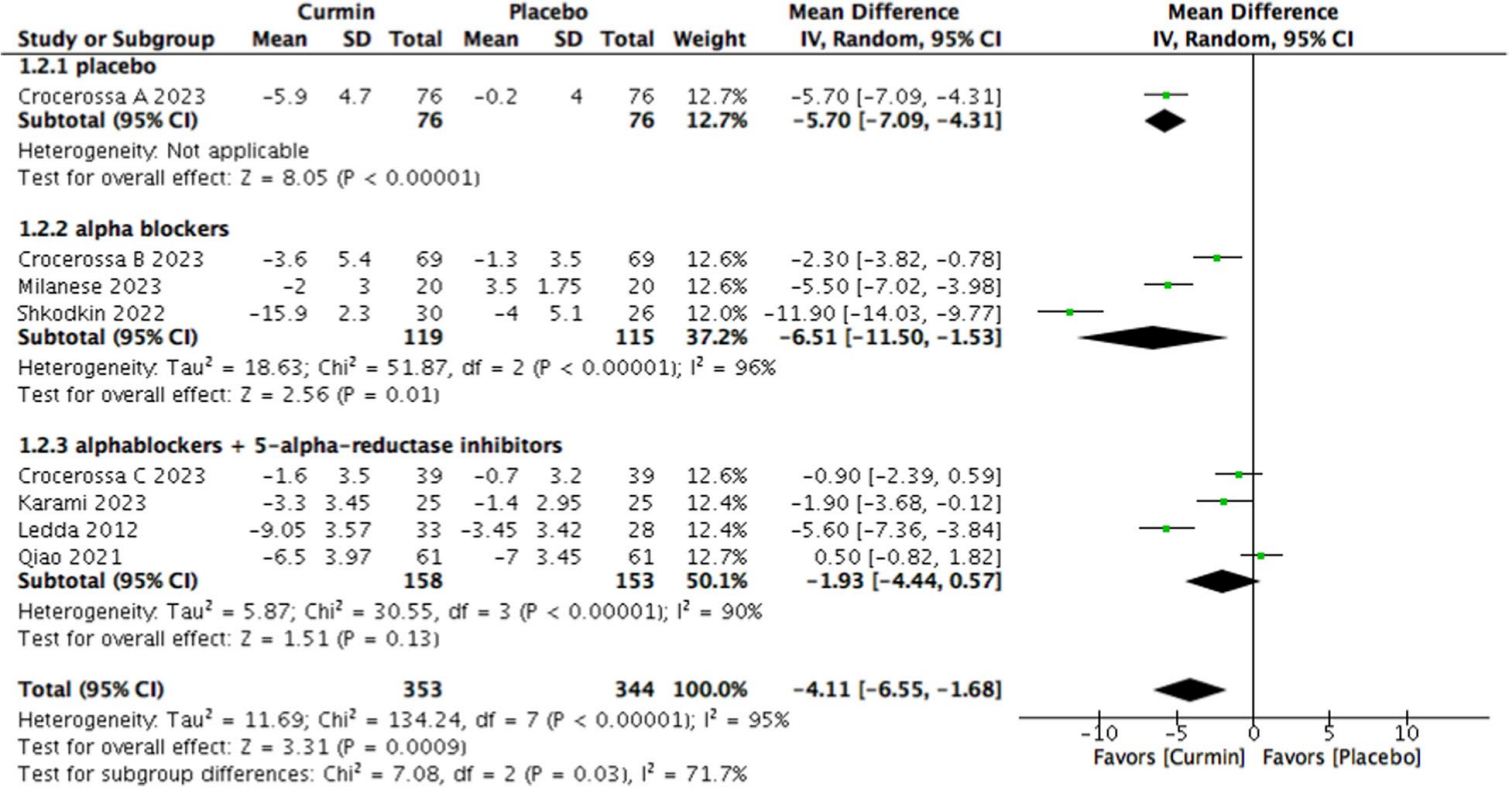
Forest plot of IPSS change comparing curcumin versus placebo

Pooled analysis demonstrated that curcumin supplementation was associated with a significant reduction in IPSS total score compared with placebo (MD − 4.11; 95% CI − 6.55 to − 1.68; *p* = 0.0009), indicating clinically meaningful symptomatic improvement (Fig. 4). Substantial heterogeneity was observed across studies (I^2^ = 95%).

**Fig. 5:**
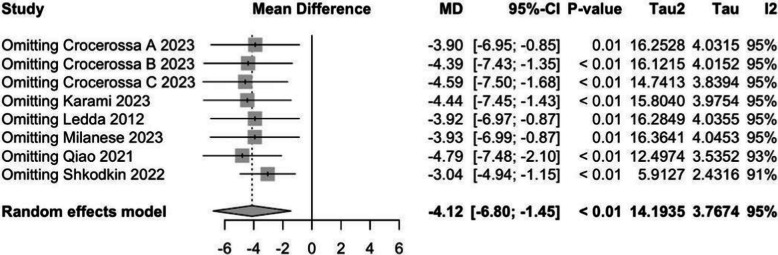
Leave-one-out sensitivity analysis for IPSS outcome

Given the high heterogeneity, leave-one-out sensitivity analyses were performed. Sequential exclusion of individual studies did not materially alter the direction or statistical significance of the pooled estimate, with mean differences ranging from − 3.04 to − 4.79 (Fig. 5), supporting the robustness of the primary outcome.

**Fig. 6:**
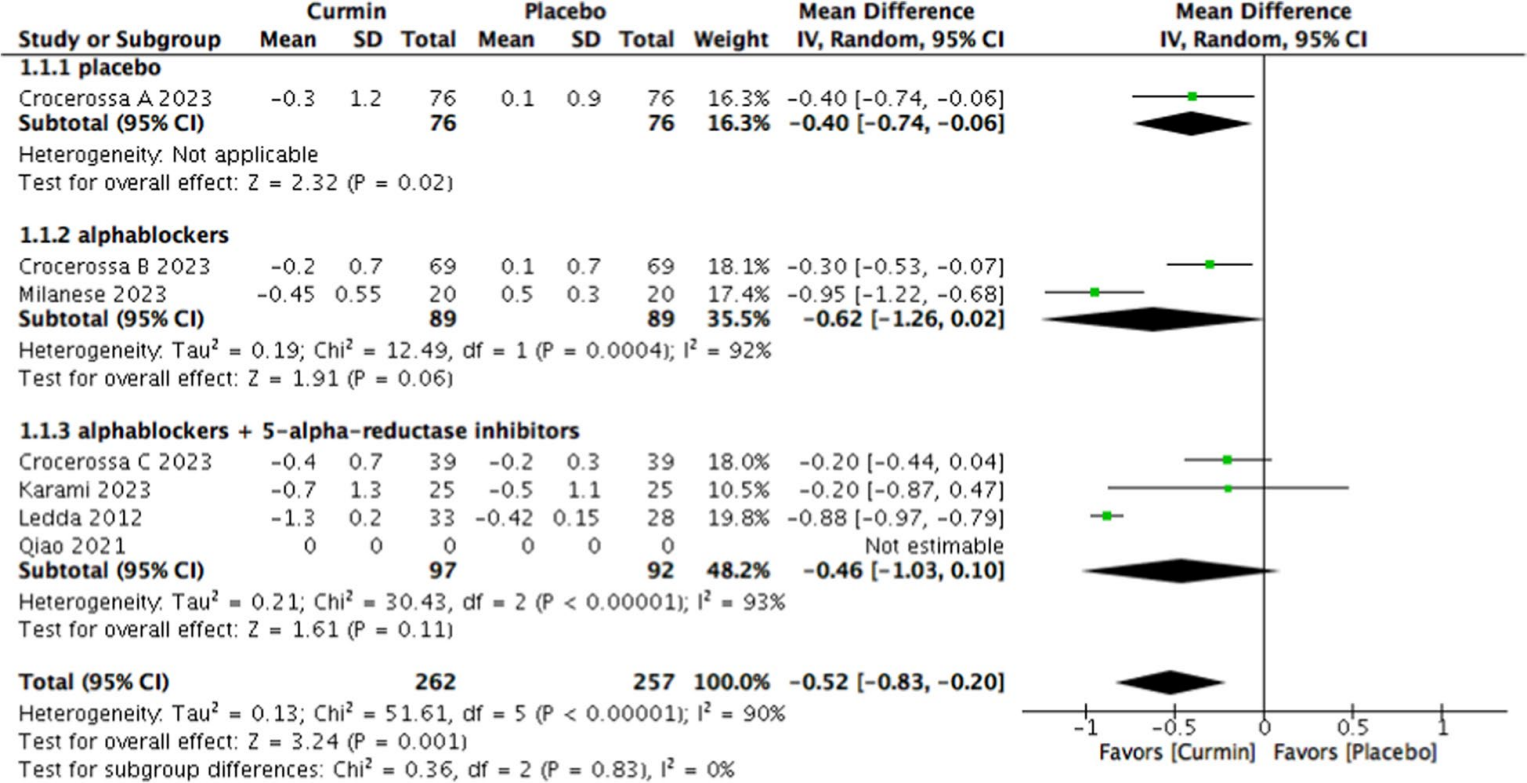
Forest plot of PSA levels comparing curcumin versus placebo

Curcumin supplementation was associated with a significant reduction in PSA levels compared with placebo (MD − 0.52 ng/mL; 95% CI − 0.83 to − 0.20; *p* = 0.001), with substantial heterogeneity among studies (I^2^ = 90%) (Fig. 6).

**Fig. 7:**
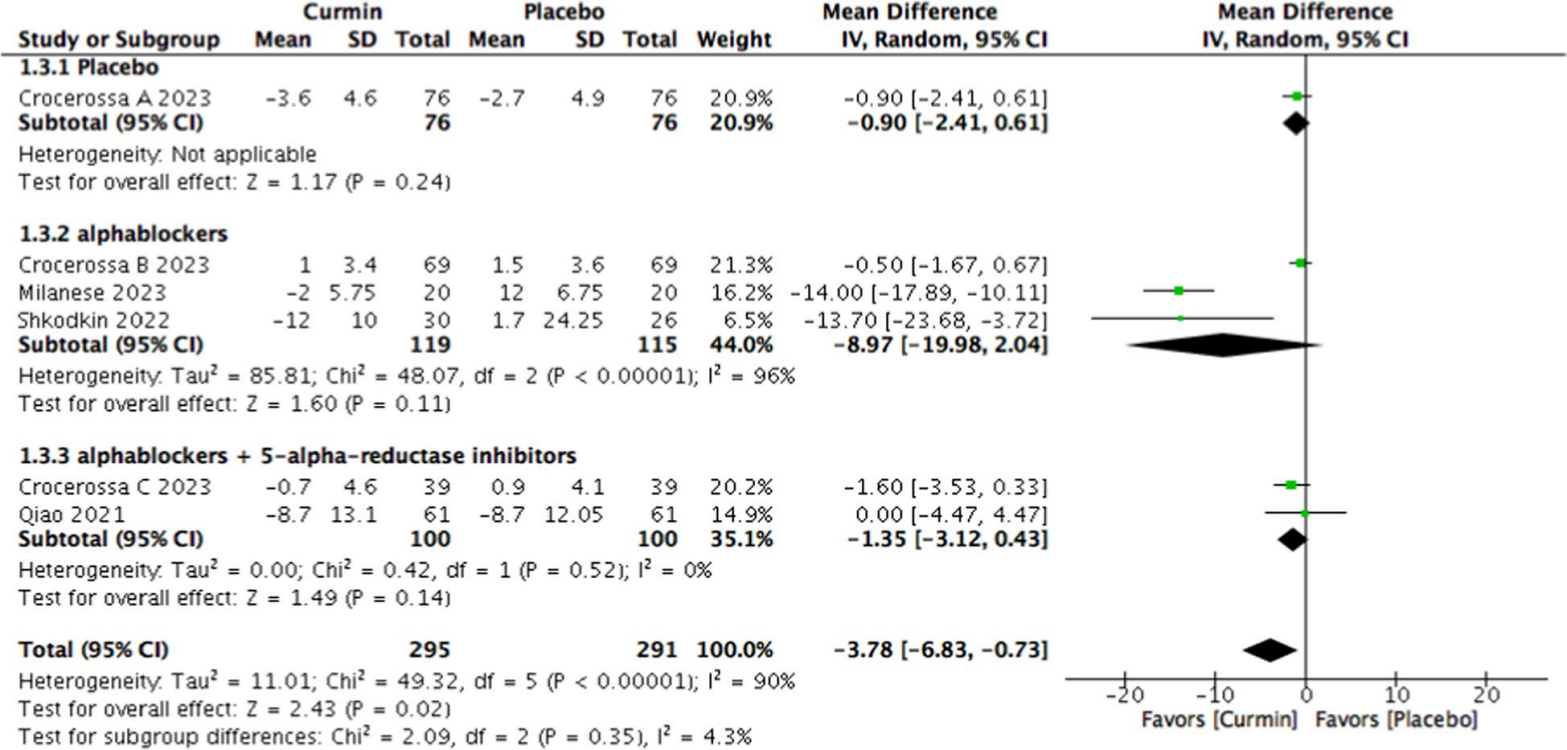
Forest plot of prostate volume comparing curcumin versus placebo

A statistically significant decrease in prostate volume was observed in the curcumin group (MD − 3.78 mL; 95% CI − 6.83 to − 0.73; *p* = 0.02); however, the magnitude of this change is likely of limited clinical relevance. Heterogeneity remained considerable (I^2^ = 90%) (Fig. 7).

**Fig. 8:**
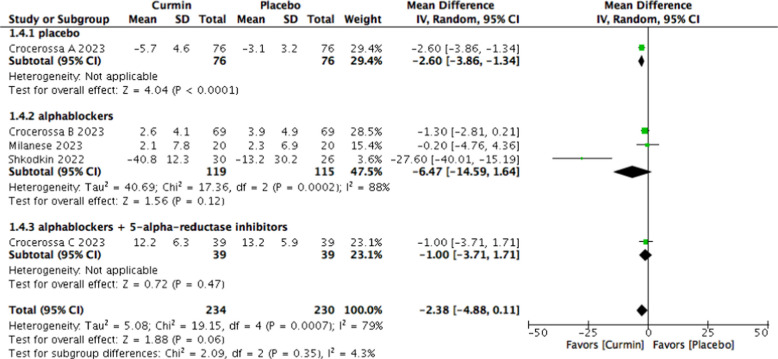
Forest plot of post-void residual volume comparing curcumin versus placebo

Curcumin use was associated with a numerical reduction in post-void residual volume; however, this did not reach statistical significance (MD − 2.38 mL; 95% CI − 4.88 to 0.11; *p* = 0.06; I^2^ = 79%) (Fig. 8).

**Fig. 9:**
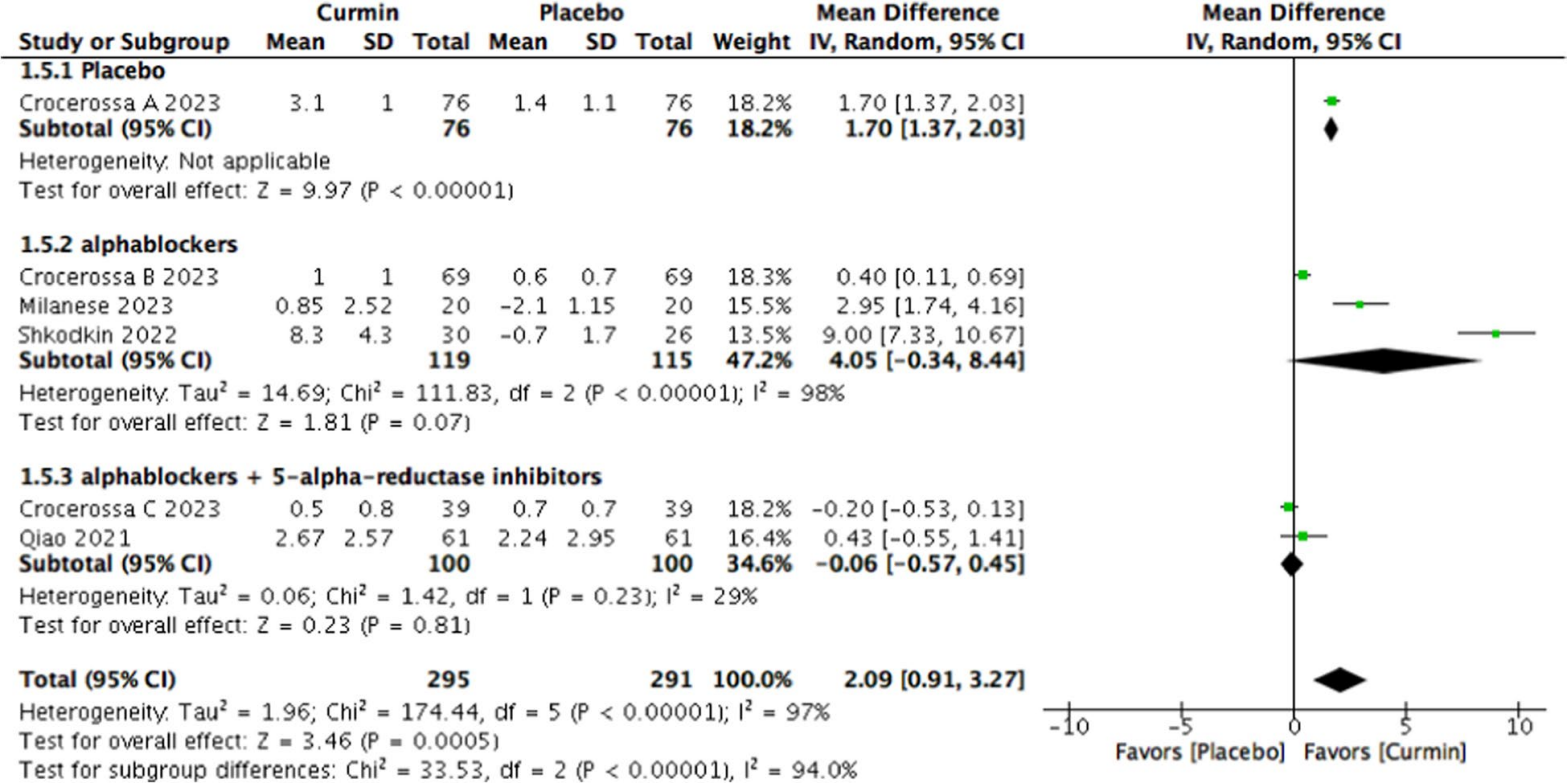
Forest plot of maximum urinary flow rate (Q-max) comparing curcumin versus placebo

Patients receiving curcumin demonstrated a significant improvement in maximum urinary flow rate compared with placebo (MD 2.09 mL/s; 95% CI 0.91 to 3.27; *p* = 0.0005), although heterogeneity was substantial (I^2^ = 97%) (Fig. 9).

Reporting of adverse events was inconsistent across studies. Overall, curcumin appeared to be well tolerated, with no significant increase in adverse events compared with placebo. However, the lack of standardized safety reporting limits definitive conclusions.

### Subgroup analyses according to background therapy

In patients receiving α1-adrenoceptor blockers alone, curcumin supplementation resulted in a significant reduction in IPSS total score (MD − 6.51; 95% CI − 11.50 to − 1.53; *p* = 0.01; I^2^ = 96%) (Fig. 4). In this subgroup, no statistically significant differences were observed in prostate volume, post-void residual volume, or maximum urinary flow rate.

In contrast, among patients receiving combined α1-adrenoceptor blocker and 5-alpha-reductase inhibitor therapy, curcumin did not significantly improve IPSS (MD − 1.93; 95% CI − 4.44 to 0.57; *p* = 0.13; I^2^ = 90%) or PSA levels (MD − 0.46 ng/mL; 95% CI − 1.03 to 0.10; *p* = 0.11; I^2^ = 93%).

Data on treatment-naïve patients were limited and inconsistently reported across studies, precluding a formal pooled subgroup analysis. However, available evidence suggests that curcumin may exert a more pronounced effect in patients not receiving concomitant pharmacological therapy.

### Risk of bias assessment

Risk-of-bias assessments are presented in Figs. 2 and 3. Among randomized controlled trials, overall risk of bias was low, with some concerns mainly related to outcome reporting. Among nonrandomized studies, two were judged to have moderate risk of bias, while one study was considered to present serious risk of bias, primarily due to confounding and participant selection domains.

## Discussion

This systematic review and meta-analysis synthesized the current clinical evidence comparing curcumin-based supplementation with placebo in men with benign prostatic hyperplasia. Across six comparative studies involving 697 patients, curcumin was associated with significant improvements in LUTS, as reflected by reductions in IPSS, alongside favorable changes in PSA, prostate volume, and maximum urinary flow. Although heterogeneity was substantial, sensitivity analyses confirmed the robustness of the symptomatic benefit, supporting a consistent direction of effect.

The observed improvement in IPSS represents the most clinically relevant outcome of this analysis. A mean reduction of approximately four points exceeds the threshold generally considered clinically meaningful and aligns with the hypothesis that targeting inflammatory pathways may offer symptomatic benefit in BPH. Unlike conventional therapies that act primarily through smooth muscle relaxation or hormonal modulation, curcumin exerts pleiotropic biological effects, including suppression of NF-κB signaling. The NF-κB pathway plays a central role in regulating inflammatory responses in prostatic tissue by controlling the transcription of cytokines such as IL-6, IL-8, and TNF-α, as well as mediators involved in oxidative stress and cellular proliferation. Chronic activation of this pathway has been associated with stromal proliferation, epithelial hyperplasia, and progression of LUTS. Curcumin inhibits NF-κB activation by preventing IκB degradation and subsequent nuclear translocation of NF-κB subunits, thereby attenuating the inflammatory microenvironment that contributes to prostatic enlargement and symptom burden. In addition, curcumin downregulates pro-inflammatory cytokine expression and modulates growth factor pathways [23, 24]. These mechanisms provide a biologically coherent explanation for the symptomatic improvement observed, particularly in a disease increasingly recognized as being driven, at least in part, by chronic inflammation [25, 26].

The reductions observed in PSA and prostate volume, although statistically significant, were modest and likely of limited clinical relevance. Similarly, changes in PVR were not clinically meaningful. These findings suggest that the primary benefit of curcumin lies in symptom modulation rather than structural modification of the prostate [27].

Subgroup analyses revealed that symptomatic benefit was more pronounced in patients receiving α1-adrenoceptor blockers alone, whereas those on combined α-blocker and 5-alpha-reductase inhibitor therapy did not demonstrate significant additional improvement. This finding may indicate a ceiling effect in patients already receiving maximal medical therapy, in whom inflammatory modulation alone is insufficient to yield further symptomatic gains [28]. Alternatively, it may suggest that curcumin’s predominant clinical impact lies in symptom modulation rather than structural prostate reduction, which is already targeted by 5-ARI therapy [29]. These findings suggest that curcumin may be better positioned as an adjunct in patients with persistent symptoms under α-blocker therapy or in those who are intolerant to standard treatments. Background therapy, particularly with 5-alpha-reductase inhibitors, represents a significant confounding factor. Given their well-established long-term effects on prostate volume and disease progression, it is difficult to isolate the independent contribution of curcumin, especially in studies with longer follow-up.

The absence of a dedicated analysis for treatment-naïve patients represents an important limitation. This subgroup is particularly relevant, as it allows for the evaluation of curcumin’s independent pharmacological effect without confounding from concomitant therapies. Future trials should prioritize stratification according to baseline treatment status.

Despite the consistency of direction across outcomes, heterogeneity was considerable. Several factors likely contributed to this variability. First, included studies encompassed both randomized and nonrandomized designs, introducing differences in baseline risk, confounding control, and outcome ascertainment. Second, curcumin formulations varied widely, ranging from conventional preparations to enhanced-bioavailability complexes such as cyclodextrin-bound and micellar formulations. Importantly, the wide variability in curcumin dosing across studies represents a major limitation for clinical translation. Third, daily dosages ranged from 75 mg to over 2,000 mg, with no clear dose–response relationship identified. Importantly, this wide variability represents a major limitation for clinical translation. Differences in formulation and bioavailability further compound this issue, precluding the identification of an optimal therapeutic dose. As a result, the applicability of these findings to clinical practice remains uncertain. Another relevant limitation is that curcumin was not consistently administered as a single agent. The frequent use of combination formulations in phytotherapy introduces potential confounding effects, making it difficult to attribute observed clinical benefits solely to curcumin.

The integration of nonrandomized studies represents both a strength and a limitation. While it increases sample size and reflects real-world practice, it also introduces confounding and selection bias. Risk-of-bias assessment revealed moderate to serious concerns in some observational studies, particularly regarding baseline differences and concomitant therapies. Consequently, although the overall findings are encouraging, they should be interpreted cautiously and not regarded as definitive evidence for routine clinical implementation.

Safety data were poorly reported across included studies, representing an important limitation. Although curcumin appeared to be well tolerated, the absence of standardized adverse event reporting precludes robust conclusions regarding its safety profile, particularly for long-term use.

Another relevant limitation is the relatively short follow-up in most trials. BPH is a chronic, slowly progressive condition, and the durability of curcumin’s effects remains uncertain. Whether symptomatic improvements are sustained beyond one year, whether structural changes translate into reduced risk of progression or acute urinary retention, and whether curcumin influences long-term treatment trajectories remain unanswered questions. Additionally, heterogeneity in follow-up duration represents a critical limitation. In patients receiving long-term therapies such as 5-ARIs, progressive improvement over time may confound the attribution of effects to curcumin, particularly in studies with longer follow-up periods.

From a clinical perspective, the present findings suggest that curcumin supplementation may represent a reasonable adjunctive strategy for selected patients with BPH, particularly those with persistent LUTS under α-blocker therapy, intolerance to conventional drugs, or preference for complementary approaches. However, its role should currently be considered exploratory rather than guideline-directed. Well-designed, adequately powered randomized trials with standardized high-bioavailability formulations, rigorous control of background therapy, and longer follow-up are necessary to define optimal dosing, identify responsive patient subgroups, and clarify its impact on disease progression.

## Conclusion

Curcumin supplementation was associated with a significant improvement in LUTS, as reflected by clinically meaningful reductions in IPSS compared with placebo. This effect was most pronounced in patients receiving α-blocker monotherapy, while no additional benefit was observed in those already receiving combined α-blocker and 5-alpha-reductase inhibitor therapy.

Overall, curcumin may represent a potential adjunctive option for symptom control in selected patients; however, its clinical applicability is limited by heterogeneity in dosing, formulations, and study design. Further well-designed randomized trials are required to define its role in BPH management.

## Supplementary Information

Below is the link to the electronic supplementary material.

Supplementary file1 (DOCX 31 KB)

## Data Availability

Available upon request.
